# Technical note: novel utilization of an H-plate for the treatment of posterior wall components of acetabular fractures

**DOI:** 10.1007/s00590-025-04544-6

**Published:** 2025-10-06

**Authors:** Anjali Malhotra, Doriann Alcaide, Robin Litten, Nigel Blackwood, Jared Halstrom, Jonathan Ellis, Clay Spitler, Joey Johnson

**Affiliations:** 1 SUNY Upstate Medical University, Syracuse, United States; 2 Department of Orthopaedic Surgery, University of Missouri–Kansas City, Kansas City, United States; 3 Department of Orthopaedic Surgery, University of Alabama at Birmingham, Birmingham, United States; 4 Department of Orthopaedic Surgery, University of Cincinnati Medical Center, Cincinnati, United States

**Keywords:** Acetabular fracture, Posterior wall fixation, H-plate, Conversion total hip arthroplasty

## Abstract

**Abstract:**

Posterior wall components of acetabular fractures are challenging injuries to treat due to the frequency of comminution, marginal impaction and high contact forces of the femoral head on the posterior wall. A variety of fixation techniques for these components have been utilized including conventional buttress plating and free screws. The objective of this report is to describe the technical application of the DePuy Synthes 2.7mm nonlocking hindfoot and midfoot plate, a 5-hole implant (the “H-plate”) (DePuy Synthes; Raynham, MA), in the surgical treatment of posterior wall fractures and to present early clinical outcomes from its use. We report a series of 95 patients who underwent fixation of posterior wall components of acetabular fractures (AO/OTA 62) with an H-plate construct. At a mean follow-up of 257 days, conversion to total hip arthroplasty occurred in six patients (6.3%), there were no postoperative dislocations, four surgical site infections (4.2%), and no cases required revision open reduction internal fixation. The H-plate’s overall shape may enhance stability due to its width and design, and these early results suggest that the H-plate may represent a viable option for stabilization of posterior wall fractures.

**Level of evidence:**

III.

## Introduction

Posterior wall components of acetabular fractures are challenging injuries to treat due to the complex anatomy of the acetabulum, as well as the frequent presence of fragment comminution and marginal impaction [1–4]. Posterior wall fractures in isolation account for approximately 25% of all acetabular fractures, with conversion rates to total hip arthroplasty (THA) reported to be as high as 28% [5]. Postoperative complications, such as post-traumatic osteoarthritis from cartilage damage, hip dislocation, and infection, contribute to this frequent conversion to THA [6].

Various implants are used to reduce and stabilize posterior wall components, including lag screws, buttress plates, rim plates, and spring plates [7, 8]. However, there is no consensus on the ideal implant for stabilization of posterior wall fragments [3, 7]. Recently, newer approaches incorporating three-dimensional templates and anatomically contoured implants have gained popularity [9, 10]. This lack of standardization in fixation strategies may contribute to variability in surgical outcomes and has driven the development of innovative techniques and novel implant utilization [11–15]. Furthermore, spring plates and reconstruction plates may provide only limited buttress strength and coverage in the setting of extensive comminution. This limitation highlights a challenge in posterior wall fixation techniques.

**Fig. 1:**
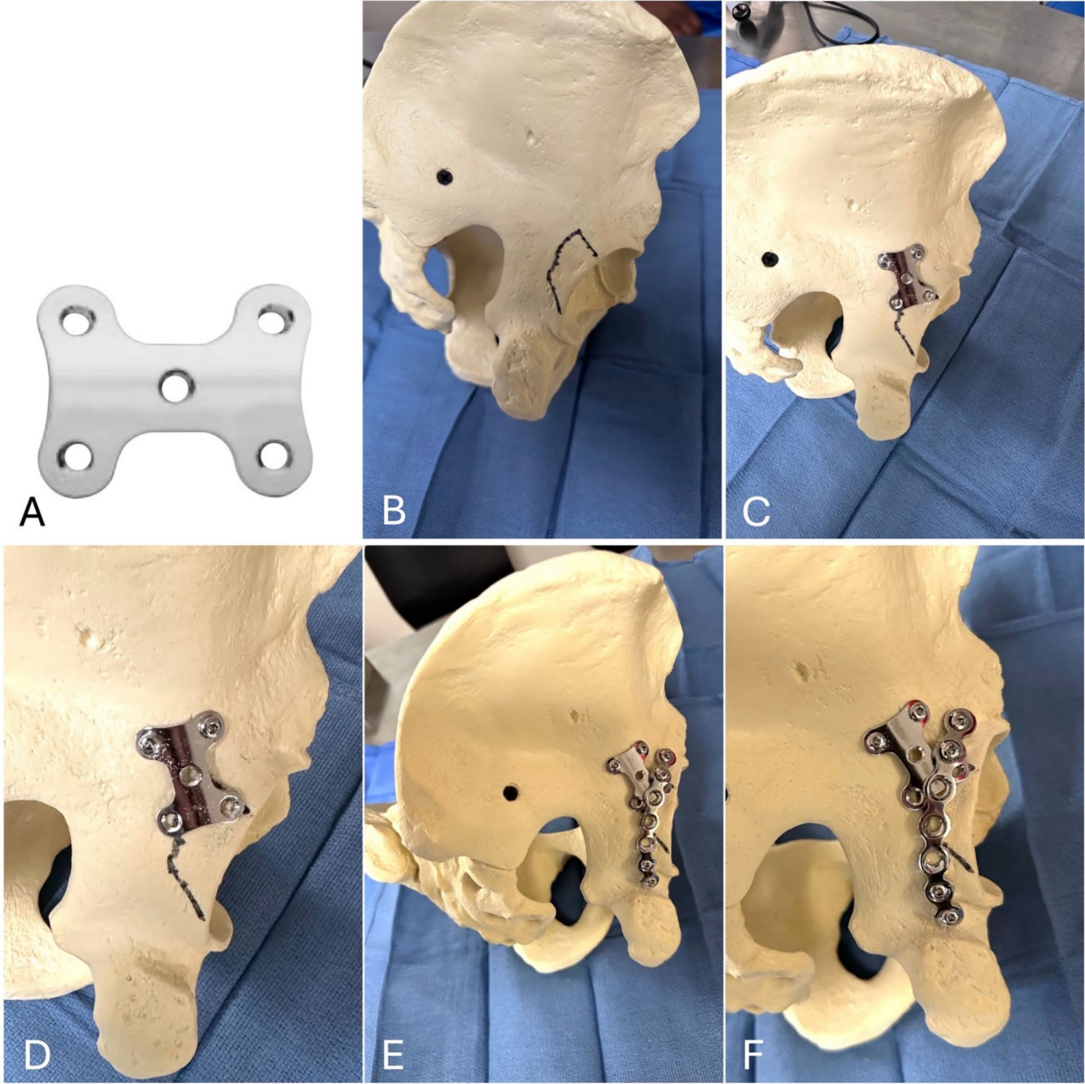
**A** 2.4 mm/2.7 mm nonlocking hindfoot/midfoot plate (DePuy Synthes; Raynham, MA), referred to as the ‘H-plate’ for its shape. **B** Posterior wall acetabular fracture illustrated on anatomic model. **C**-**D** H-plate positioned over the posterior wall fracture with two screws inserted medially. **E**–**F** Contoured 7-hole reconstruction plate secured over H-plate with four screws, two proximal and two distal to the fracture line

The DePuy Synthes 2.7 mm nonlocking hindfoot and midfoot plate, a 5-hole implant referred to as the ‘H-plate’ (DePuy Synthes; Raynham, MA), has been identified as a useful option for addressing some of the challenges in the surgical treatment of posterior wall acetabular fractures (Fig. 1). While developed for hindfoot and midfoot applications, this 5-hole nonlocking plate has shown potential for use in posterior wall acetabular fixation. Its distinct width, shape, and increased stiffness compared with traditional spring plates allow it to buttress a larger surface area and may provide improved stability in comminuted fracture patterns. This novel application of the H-plate therefore represents an innovative adaptation of an existing implant to address a limitation in acetabular fracture fixation.

Within this cohort, the H-plate was utilized for treating posterior wall components of acetabular fractures for its ease of application, ability to buttress a broad surface area, and improved implant stiffness in comparison to traditional spring plates. This report describes a novel technique for fixation of posterior wall acetabular fractures (AO/OTA 62) using the H-plate and presents early clinical outcomes from a series of 95 patients who underwent operative fixation using this technique.

## Operative technique

**Fig. 2:**
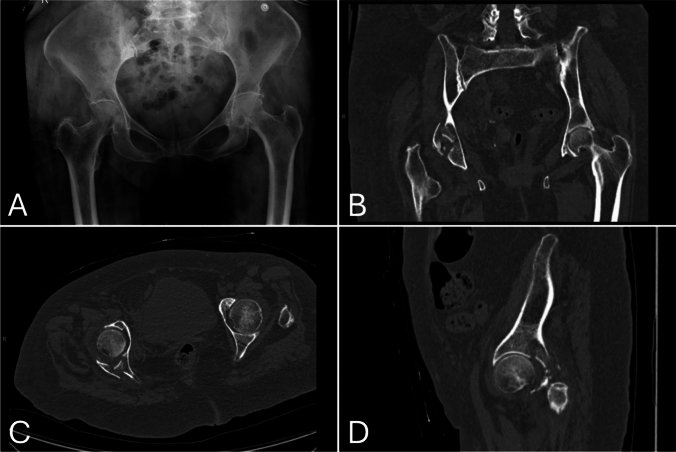
Anteroposterior (AP) radiograph of the pelvis **A** and coronal **B**, axial **C**, and sagittal **D** computed tomography (CT) images of the right hip obtained upon presentation to the emergency department following a ground-level fall. Imaging demonstrates a posterior column and posterior wall acetabular fracture

We present the case of an 84-year-old female who sustained a posterior column with posterior wall acetabular fracture following a ground-level fall. Advanced imaging confirmed the fracture pattern (Fig. 2).

### Setup

The patient is positioned on a radiolucent operating table in either the lateral decubitus or prone position, depending on surgeon preference and fracture pattern, with bony prominences carefully supported using extra padding. In this case, the patient was positioned in lateral decubitus.

### Approach

A standard Kocher-Langenbeck approach was made by identifying the posterior superior iliac spine and the greater trochanter and then extended distally in line with the femoral shaft. After incising the skin, sharp dissection was used to split the iliotibial (IT) band and the gluteus maximus was then divided in line with the IT band splint. The short external rotators were identified and tagged with a suture. These were cut approximately a centimeter from the insertion site on the femur. The sciatic nerve was identified and was protected through the operation by keeping the hip extended and knee flexed. Any injured portion of the gluteus minimus was then debrided as a prophylactic measure for heterotopic ossification.

### Reduction and stabilization

Once the retroacetabular surface was visualized, the posterior wall fracture was booked open to further inspect the joint surface. The hip was distracted, and the joint surface was inspected for loose bodies and marginal impaction. Any loose fragments within the hip joint were identified and removed to allow for a concentric reduction. Given the marginal impaction present in this patient, this was then elevated back to the femoral head using osteotomes and tamps until it was concentrically reduced. A calcium phosphate bone void filler was then used to support the impacted area. Independent fixation was not placed into the reduced articular segment because of its narrow width.

**Fig. 3:**
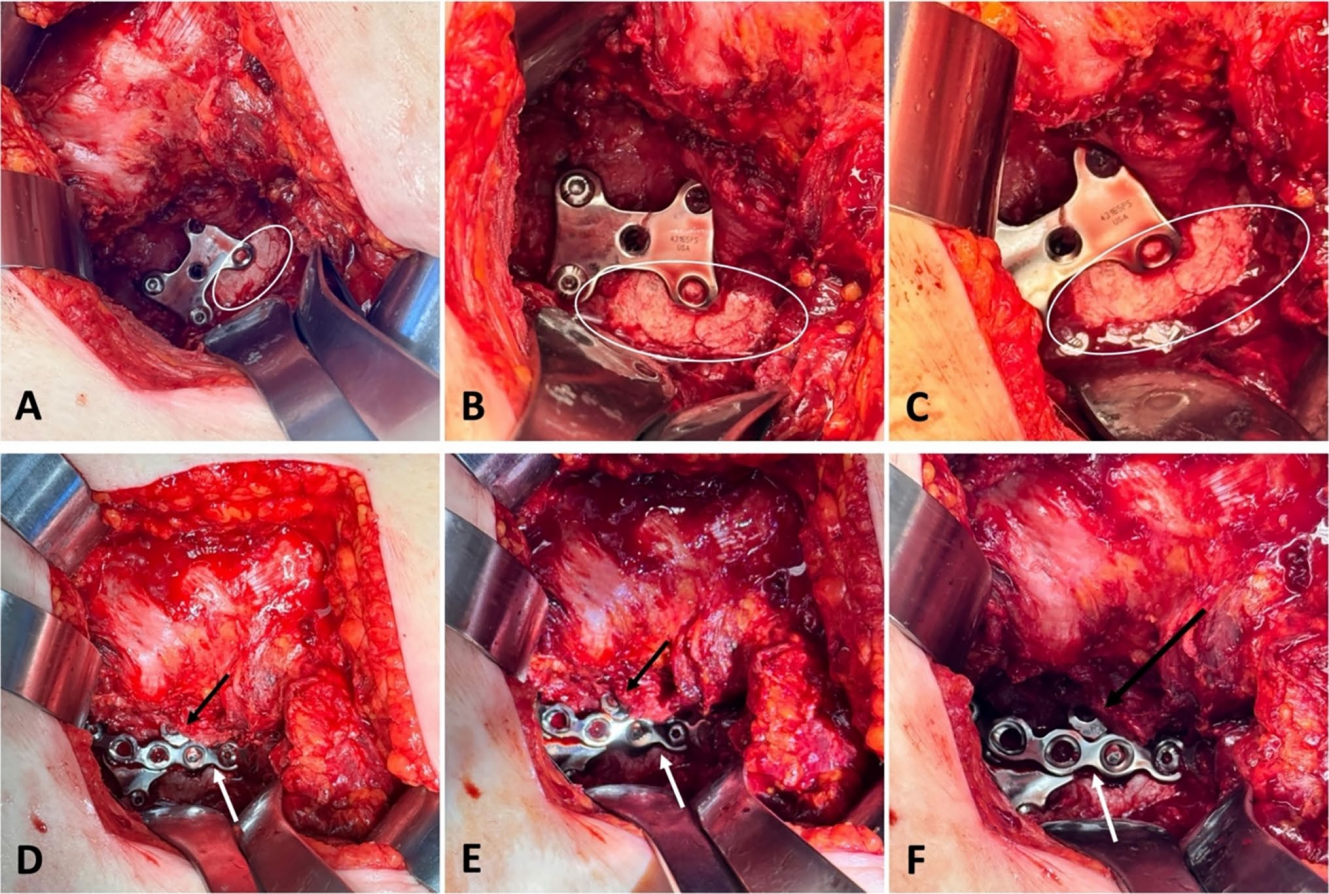
Intraoperative photographs demonstrating fixation of a posterior wall component of an acetabulum fracture using an H-plate construct. **A**-**C** The H-plate positioned to buttress the posterior wall fragment for primary stabilization with void filler (white circle) positioned adjacent to the H-plate as there was cortical comminution that was not reconstructable associated with marginal impaction. **D**-**F** A 7-hole reconstruction plate (white arrow) contoured and applied over the H-plate (black arrow) to provide supplemental fixation for an associated column fracture and buttress support of the posterior wall

The posterior wall component of the fracture was then cleaned with a #15 blade and the fracture was reduced with a ball spike or fracture reduction clamp and temporarily stabilized using Kirschner (K) wires. There was also an area of cortical impaction, likely from a femoral head dislocation, that was not reconstructable, and the same calcium phosphate bone void filler was used to fill this cortical defect. At this point, the H-plate was positioned onto the posterior wall fragment and secured using two 3.5 mm cortical screws placed medial to the fracture site, as demonstrated on both an anatomic model and intraoperative photographs (Fig. 1C, D; Fig. 3A–C). The H-plate was applied without contouring.

**Fig. 4:**
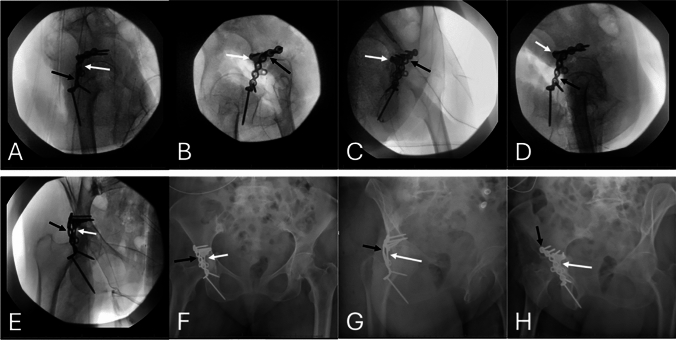
Intraoperative fluoroscopic images **A**-**E** demonstrating reduction and fixation of a posterior wall acetabular fracture. The H-plate (white arrows) was positioned to buttress the posterior wall fragment for primary stabilization. A 7-hole reconstruction plate (black arrows) was contoured and applied over the H-plate (white arrows) to provide supplemental fixation for an associated column fracture and buttress support of the posterior wall. **F**–**H** Postoperative radiographs of the pelvis, including anteroposterior (AP) view **F**, obturator oblique view **G**, and iliac oblique view **H**. Images demonstrate appropriate hardware positioning, restoration of posterior column alignment, and joint congruity following fixation of the posterior wall acetabular fracture

The shape of the H-plate provides a buttress effect similar to that of a spring plate but distributed over a larger surface area and through a stiffer implant which allows greater compression. The posterior column component of the fracture was further reduced and stabilized with a contoured 7-hole reconstruction plate placed over the H-plate, as demonstrated on both an anatomic model and intraoperative photographs (Fig. 1E, F; Fig. 3D–F). This helped secure and further buttress the H-plate. This reconstruction plate was held in place with a total of four 3.5 mm cortical screws, two proximal and two distal to the posterior column and posterior wall fracture lines. The reduction was then confirmed with intraoperative fluoroscopy, including anteroposterior (AP), obturator oblique, and iliac oblique views of the pelvis (Fig. 4).

### Closure and postoperative care

After completion of fixation, the short external rotators were repaired through drill holes in the greater trochanter. The incisions were irrigated and closed in layers. Sterile dressings were applied intraoperatively. The patient was instructed to remain non-weight bearing on the right lower extremity, to perform range of motion as tolerated, and to continue deep vein thrombosis prophylaxis until weightbearing is permitted. Immediate postoperative radiographs are shown in Fig. 4.

## Case series

This case series includes 95 consecutive patients who underwent operative fixation of posterior wall acetabular fractures with an H-plate. In all cases, an H-plate was utilized in combination with a supplemental buttress plate. The mean patient age was 42.7 years (range: 19–84 years), with a predominance of males (*n* = 67, 70.5%) (Table 1). All patients in this cohort had a follow-up to bony union, with a mean follow-up of 257.0 days (range: 83–796 days) (Table 1). Bony union was assessed at routine follow-up using standard anteroposterior and oblique pelvic radiographs. Union was defined as radiographic evidence of bridging callus across the fracture site without hardware failure, as determined by the treating orthopaedic trauma surgeons [2, 16].

**Table 1:** Demographic, clinical, and injury characteristics among 95 patients with posterior wall acetabular fractures

Demographic & clinical characteristics	Frequency (%) or mean (SD)
Age	42.7 (15.7)
Follow-up (days)	257.0 (154.2)
*Sex*	
Male	67 (70.5)
Female	28 (29.5)
Body mass index (kg/m^2^)	31.7 (9.1)
Tobacco use	38 (40.0)
Diabetes	15 (15.8)
Hypertension	21 (22.1)
Alcohol use	18 (18.9)
*ASA score*	
1	2 (2.1)
2	27 (28.4)
3	53 (55.8)
4	13 (13.7)
*Injury characteristics*	
Injury severity score	23.5 (24.6)
*Mechanism of injury*	
ATV	1 (1.1)
Crush	1 (1.1)
FFH	5 (5.3)
FFS	4 (4.2)
MCC	7 (7.4)
MVC	77 (81.1)
*Letournel classification*	
PC PW	9 (9.5)
PW	44 (46.3)
T-type + PW	0
TPW	42 (44.2)
Marginal impaction	10 (10.5)
Femoral head injury	13 (13.7)
Percent of posterior wall involvement*	
< 20%	6 (6.3)
20–50%	31 (32.6)
> 50%	58 (61.1)
Quality of reduction**	
Anatomic	70 (73.7)
Imperfect	20 (21.1)
Poor	5 (5.3)

SD = standard deviation, kg/m^2^ = kilograms per meter squared, ASA = American society of anesthesiologists. ATV = all-terrain vehicle accident, crush = crush injury, FFH = fall from height, FFS = fall from standing, MCC = motorcycle collision, MVC = motor vehicle collusion, PC PW = posterior column with posterior wall fracture, PW = posterior wall fracture only, T-type + PW = T-type fracture combined with posterior wall fracture, TPW = transverse with posterior wall fracture

^*^Posterior wall measurements were obtained using the method described by Moed et al. [17, 18]

^**^Defined using Matta’s criteria [16]

The mean body mass index (BMI) of this cohort was 31.7 kg/m^2^ (Table 1). Tobacco use was documented in 38 patients (40.0%), alcohol use in 18 (18.9%), hypertension in 21 (22.1%), and diabetes in 15 patients (15.8%). The majority of patients were classified as American Society of Anesthesiologists (ASA) class III (Table 1).

The mean Injury Severity Score (ISS) was 23.5 (Table 1). The most common mechanism of injury was motor vehicle collision (MVC), accounting for 77 cases (81.1%) (Table 1). Other mechanisms included motorcycle collisions (7 cases, 7.4%), falls from height (FFH; 5 cases, 5.3%), falls from standing (FFS; 4 cases, 4.2%), all-terrain vehicle (ATV) accidents (1 case, 1.1%), and crush injuries (1 case, 1.1%) (Table 1).

Fracture type based on the Letournel classification system included posterior wall (PW) fractures in 44 patients (46.3%), transverse with posterior wall (TPW) in 42 (44.2%), and posterior column with posterior wall (PC PW) in 9 (9.5%) (Table 1). No T-type fractures with posterior wall involvement were observed in this cohort. Marginal impaction was identified in ten patients (10.5%), and an associated femoral head injury was noted in 13 (13.7%). Posterior wall involvement, categorized by percentage of wall affected, was < 20% in six patients (6.3%), 20–50% in 31 patients (32.6%), and > 50% in 58 patients (61.1%), using thresholds previously described by Moed et al. [17, 18]. Quality of fracture reduction, defined by Matta’s criteria [16], was classified as anatomic in 70 patients (73.7%), imperfect in 20 (21.1%), and poor in 5 (5.3%) (Table 1).

Table 2 summarizes the clinical outcomes following H-plate fixation in this cohort. Conversion to total hip arthroplasty (THA) was required in 6 patients (6.3%), with no cases necessitating revision open reduction internal fixation (ORIF). Other unplanned reoperations occurred in 3 patients (3.2%). Surgical site infections were reported in 4 patients (4.2%), and no postoperative dislocations were documented. Mortality was observed in 2 patients (2.1%) from unrelated causes during the follow-up period.

**Table 2:** Clinical outcomes following H-plate fixation of posterior wall acetabular fractures in 95 patients

Outcome	Frequency	Percentage
THA conversion	6	6.3
Revision ORIF	0	0
Other unplanned reoperation	3	3.2
Surgical site infection	4	4.2
Dislocation	0	0
Mortality	2	2.1

*THA*  total hip arthroplasty, *ORIF* open reduction internal fixation

## Discussion

Posterior wall components of acetabular fractures are associated with high complication rates due to their complex anatomy and high contact forces [2, 4, 19]. These factors, as well as surgical complications, can contribute to early THA. The H-plate has been utilized as an alternative fixation method aiming to enhance stability while minimizing complications and lowering the risk of conversion to total hip arthroplasty.

Techniques using spring plates and reconstruction plates can limit the ability to adequately buttress independent fragments, whereas the larger H-plate spans more of the retroacetabular surface, offering broader coverage of posterior wall fracture fragments, a higher level of compression via buttressing and improved structural support. Its unique shape, with increased depth and width, allows for a greater area of the posterior wall to be buttressed, and the increased plate thickness as compared to spring plates may allow for a stiffer fixation construct. Additionally, there are cases in which the medial holes can be positioned to allow for initial fixation for the transverse components of transverse posterior wall fractures that allows clamp removal during application of additional fixation. The peripheral holes in the plate can also function as an anchor point for braided suture that is at times used to augment fracture fixation for capsular or labral repair, though this application was not systematically evaluated in our series. While these design features suggest potential mechanical advantages, biomechanical studies are needed to confirm this.

Among the 95 patients in this series, 6 (6.3%) required conversion to THA. This rate is notably lower than the reported averages (up to 28%) seen across various fixation techniques for acetabular fractures [20, 21]. While not a direct comparison, Lee et al. reported a 7.7% conversion rate to THA in a cohort of 52 patients with posterior wall fractures treated using spring plates [22]. Similarly, Chuang et al. observed a 15% conversion rate among patients whose fractures were managed with reconstruction plates [23]. Although differences in cohort size, mean follow-up duration, fracture patterns, and surgical techniques limit direct comparison, these findings suggest that the H-plate may be a viable option for fixation of posterior wall fractures. The majority of patients in our cohort had > 50% posterior wall involvement. However, we also treated patients with fractures spanning < 20% of the posterior wall, suggesting that the H-plate may be a versatile implant capable of providing mechanical support in a variety of posterior wall fragment sizes.

Patients in this cohort demonstrated notable baseline comorbidities, including a mean BMI of 31.7 kg/m^2^ and tobacco use documented in 40.0%. These risk factors typically increase the likelihood of conversion to THA, suggesting that the observed outcomes in this case series may underestimate the effectiveness of H-plate fixation [21, 24]. Notably, there were no documented cases of revision ORIF or postoperative hip dislocation in this cohort, despite the majority of fractures involving > 50% of the posterior wall. This suggests that the H‑plate may provide mechanical support even in cases with significant posterior wall compromise.

This case series demonstrates that the H-plate may offer advantages in fixation of posterior wall fractures. There are several features of the H-plate’s design that may contribute to improved stabilization of posterior wall fragments, allow for an easier transition from clamp application to definitive fixation in transverse posterior wall fractures, and can serve as a nearly anatomic anchor site for capsule and labral repairs. Its application may enhance postoperative outcomes in the treatment of these challenging injuries.

### Limitations

This study has limitations that should be considered when interpreting these findings, including those inherent to its retrospective design. This study was conducted at a single institution, which may limit generalizability to other settings with different patient populations, clinical protocols, or acetabular fracture management practices. The discretionary use of the H-plate may have led to its preferential application in patients with greater comorbidity burden or more complex fracture cases, which could have affected clinical outcomes. Additionally, limitations in patient follow-up may have restricted the ability to fully assess conversion to total hip arthroplasty and post-traumatic arthritis in this cohort. This study does not include a comparator group using spring plates or reconstruction plates, which limits direct conclusions about the relative advantages of the H-plate. Although the H-plate’s broader surface area and increased stiffness suggest potential mechanical advantages over spring plates, no biomechanical studies of this implant have been published to date. Future biomechanical testing and comparative clinical studies are warranted to validate these proposed benefits.

## Conclusion

The H-plate may serve as a viable option for fixation of posterior wall acetabular fractures, particularly in cases with extensive comminution, and in this series was associated with zero postoperative dislocations. Its shape and strength may provide improved stability in these complex fracture patterns. Future studies are warranted to validate these findings.

## Data Availability

No datasets were generated or analysed during the current study.
